# Electrical effect on probe wounds after long time exposure with a new generation conducted electrical weapon (Taser T10®) on human volunteers

**DOI:** 10.1007/s12024-025-01025-4

**Published:** 2025-06-21

**Authors:** S. N. Kunz, J. D. Ho, D. M. Dawes, J. R. Miner

**Affiliations:** 1 Institute of Forensic Medicine, Ulm University, Prittwitzstr. 6, 89075 Ulm, Germany; 2 Dept. of Emergency Medicine, Univ. of Minnesota Medical School, 701 Park Ave S, Minneapolis, MN 55415 USA; 3 Exer Urgent Care, 359 Carmen Drive, Santa Barbara, CA 93010 USA

**Keywords:** Forensic medicine, TASER, Conducted electrical weapon, Wound ballistic, Probe, Long duration electrical exposure

## Abstract

Conducted electrical weapons (CEWs) are widely employed by law enforcement agencies globally to manage and restrain potentially violent individuals. As newer generations of these weapons are introduced, it is crucial to assess their effectiveness and safety profiles. The TASER 10 (T10) represents a significant advancement, utilizing an independently targeted probe system with floating polarities, enabling any two probes to establish a connection. This design enhances its versatility and operational effectiveness compared to earlier models. In scenarios requiring use over greater distances— such as deployment via drones—prolonged electrical exposure may be necessary to bridge the gap to the subject and secure control of the situation. The extended exposure duration associated with these probes may result in altered wound mechanisms and morphological features. This study examines the morphological wound characteristics of 20–30 s of electrical exposure delivered through hand-placed dart electrodes of the T10 CEW. To our knowledge, this represents the first human study to investigate potential electrical injuries resulting from extended-duration exposure with the T10 CEW.

## Introduction

Handheld conducted electrical weapons (CEWs) have been widely utilized by law enforcement and military personnel globally since their introduction in the late 1990 s. With over 1,000 publications, CEWs represent the most extensively studied force option available to law enforcement agencies. These devices function by transmitting specific electrical waveforms through at least two probe electrodes that penetrate the skin upon impact. The electrical field generated between the two electrodes (anode and cathode) stimulates peripheral motoneurons, causing involuntary muscle contractions that incapacitate the target for the duration of the deployment.

In early 2023, Axon Enterprise, Inc. (Scottsdale, AZ, USA) released a next-generation CEW, the TASER 10 (T10). Unlike earlier models, the T10 deploys single (unpaired) darts with each trigger pull, guided by a laser aiming mechanism that allows for individual dart placement. Additionally, the T10 darts are launched at higher muzzle velocities using a black powder primer ignition system, designed to improve penetration through clothing. The initial production velocity for the T10 dart was 53.3 m/s ± 9.1 m/s (175 ft/s ± 30 ft/s); however, after initial evaluation, this velocity was increased to 62.5 m/s ± 9.1 m/s (205 ft/s ± 30 ft/s) to further enhance clothing penetration capabilities.

In situations where the T10 device is used over greater distances—such as deployments via drones—longer electrical exposure durations may be required to maintain control of the subject until officers can intervene. A usual exposure lasts for 5 s. If necessary, officers can extend the exposure by pulling the trigger and thus adding additional cycles of 5 s exposure. However, these extended exposure durations may alter the morphological characteristics of the injury.

Given the design differences and the expanded operational applications of the T10, it is critical to evaluate its safety profile, particularly regarding potential electrical injuries to human tissue. The objective of this study was to assess the potential for electrical injuries caused by the T10 darts during extended exposure durations of 20–30 s. To our knowledge, this is the first human study to investigate the morphological characteristics of electrical injuries resulting from prolonged exposure to the T10 CEW.

## Materials and methods

This prospective observational study was conducted with human volunteers participating in an Axon Conducted Electrical Weapon (CEW) training course. Ethical approval for the study was obtained from the Institutional Review Board (IRB) at Hennepin County Medical Center (Minneapolis, MN). A total of 14 adult human volunteers were enrolled in the study and underwent planned exposures to the TASER 10 (T10) CEW. Prior to participation, all subjects provided informed consent for T10 dart deployments consisting of two darts applied to bare skin.

## Study population

Participants were recruited as a convenience sample on a first-come, first-enrolled basis. The sole exclusion criterion was pregnancy, which was assessed through self-reporting by prospective participants. Enrolled subjects completed a medical screening questionnaire, which was reviewed by a study physician, and had the opportunity to discuss any medical concerns or questions with the physician. Participants received an Axon CEW and monetary compensation of $500 for their participation.

## Study design

**Fig. 1:**
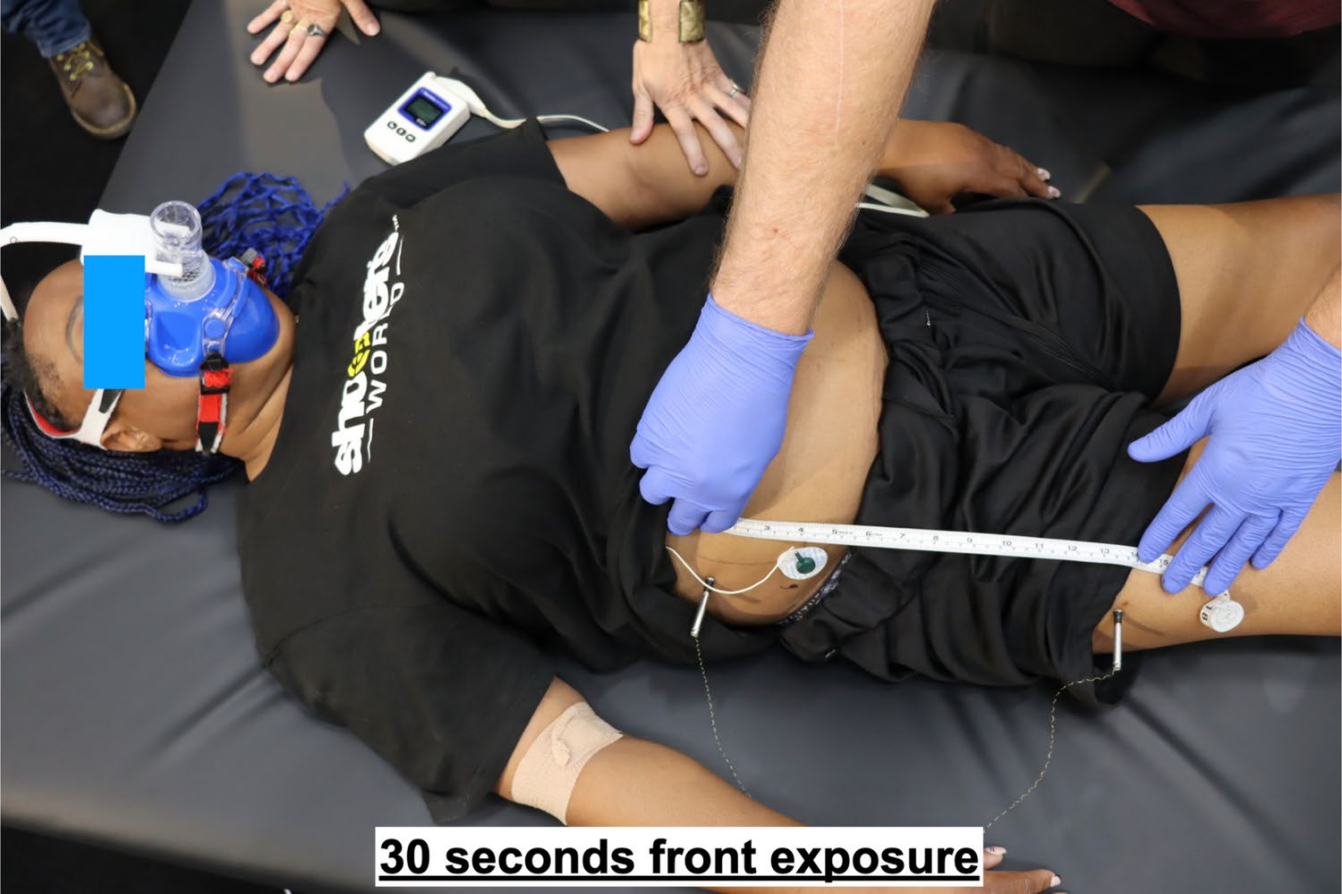
Dart placements on the frontal side

**Fig. 2:**
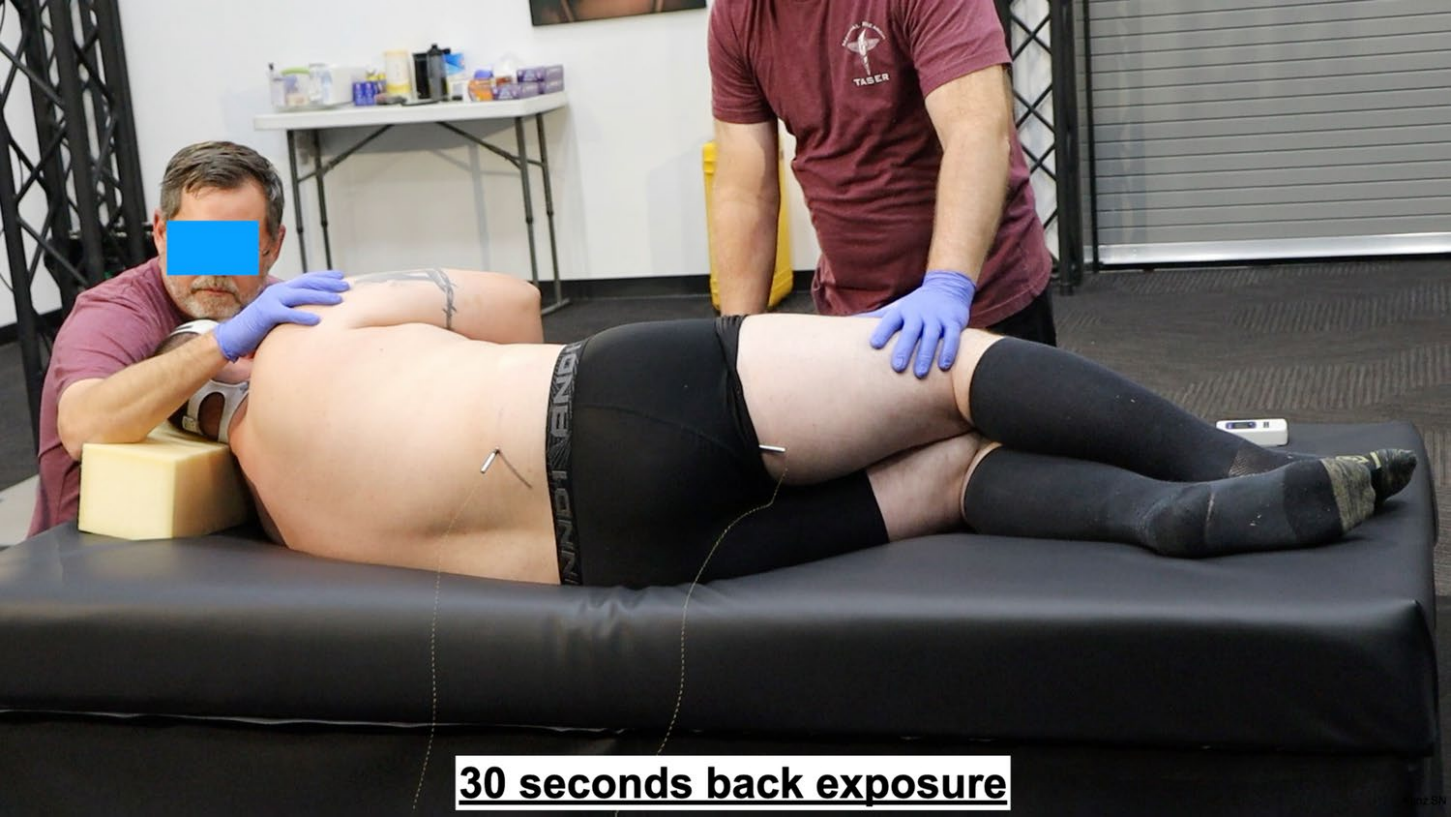
Dart placements on the back side

**Fig. 3:**
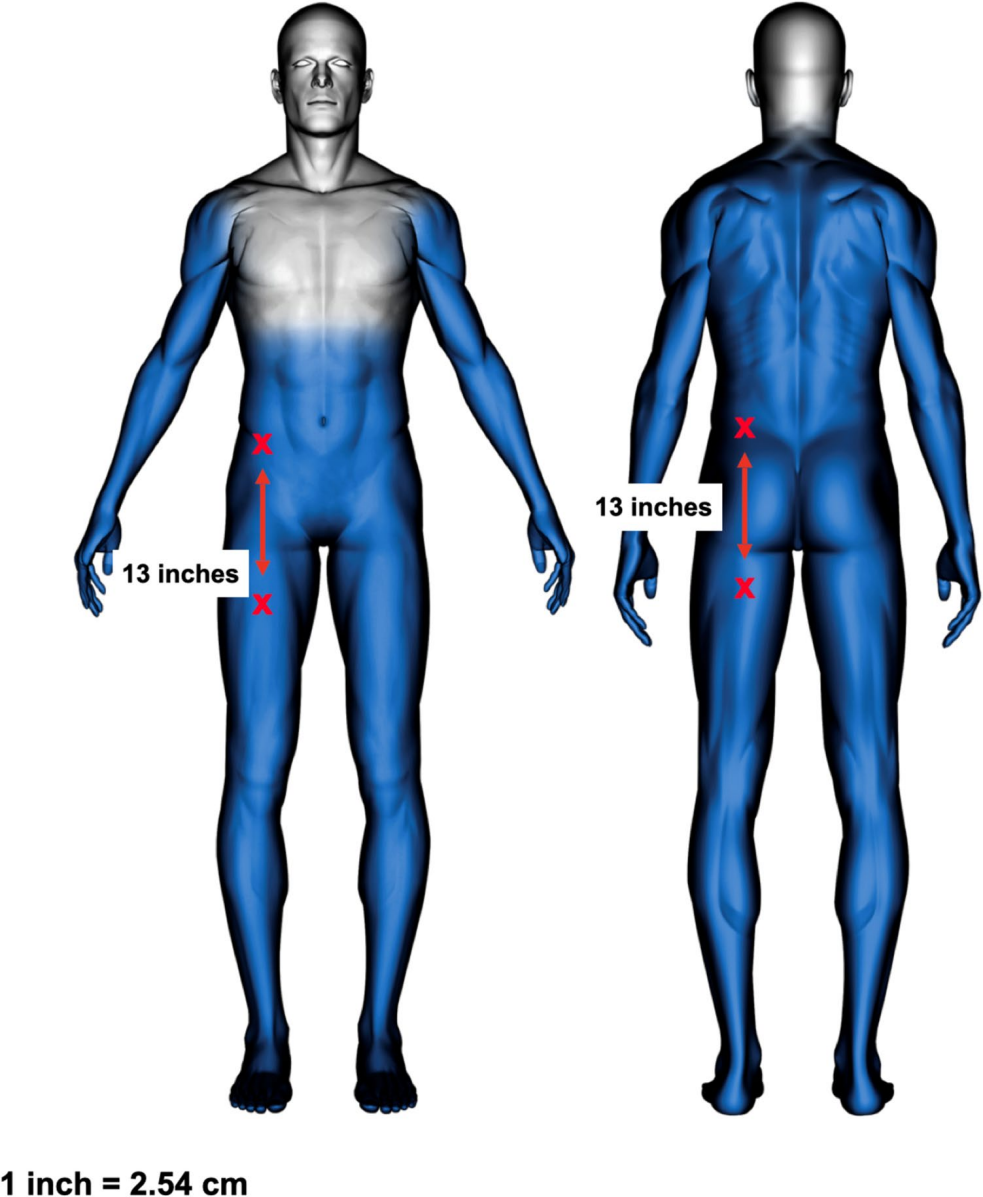
Dart placements in both groups

The study participants were sequentially assigned into two exposure groups, as detailed in Table 1. Each participant was subjected to a single exposure to the electrical waveform of the T10 CEW for a duration of at least 20 s, with a maximum duration of 30 s. The exposure site was either the lower frontal (Fig. 1) or lower posterior side (Fig. 2) of the body, depending on group assignment (Fig. 3). Subjects were provided with an emergency button that allowed them to terminate the exposure voluntarily after 20 s if desired. If not terminated manually, the exposure ended automatically at the 30-s mark.

**Table 1:** Participant characteristics

	**M/F**	**Age**	**BMI**	**Exposure site**	**Exposure time (s)**
	F	36	28.4	back	20.1
	F	23	24.2	front	28.8
	F	32	24.1	front	20.2
	M	25	41.0	front	22.1
	M	23	26.6	back	29.9
	F	43	37.1	front	19.9
	M	33	43.1	back	29.9
	M	19	17.4	back	20.7
	M	51	35.2	front	20.0
	M	30	33.4	back	29.9
	F	45	30.5	front	29.9
	M	47	27.5	back	29.9
	M	27	46.8	front	29.9
	M	39	40.0	back	29.9
	5 F/9 M	∅ 33.79	∅ 32.52	7f/7b	∅ 25.79

## Procedure

T10 dart deployments were conducted under controlled conditions, with darts hand-placed to ensure proper positioning and safety (Fig. 1). The T10 device was used according to manufacturer specifications, and the electrical waveform parameters were documented for each exposure. Morphological and clinical observations of the exposure sites were recorded pre and immediately and 24 h post-exposure to evaluate the potential for electrical injuries.

## Results

A total of 14 participants (9 males and 5 females) completed the study. Their age average was 33.8 years old, with an average BMI of 32.5. The exposition site was evenly distributed between the front of the body (7 times) and the back of the body (7 times). The study was aborted by 6 volunteers after 20–25 s. One participant stopped the exposure after 28.8 s. 7 subjects completed the full 30 s exposure (Table 1).

No complications were observed, apart from minor bleeding at the probe insertion sites.

**Fig. 4:**
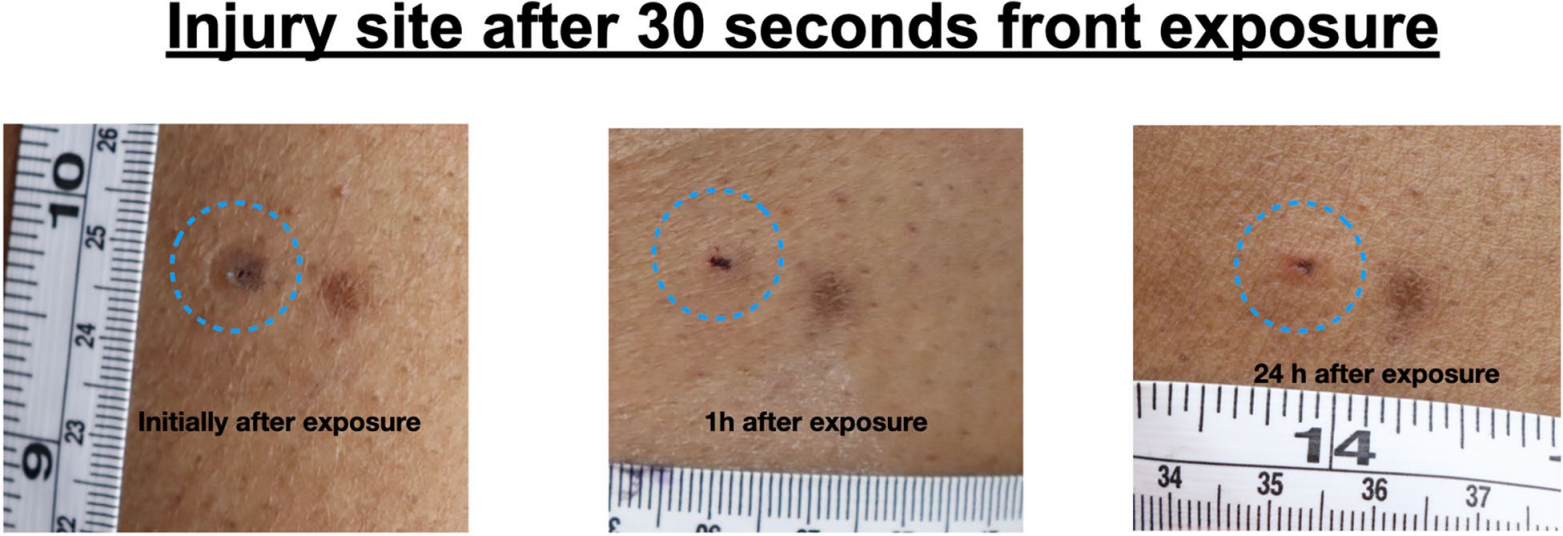
Injury site after 30 s front exposure with a T10

**Fig. 5:**
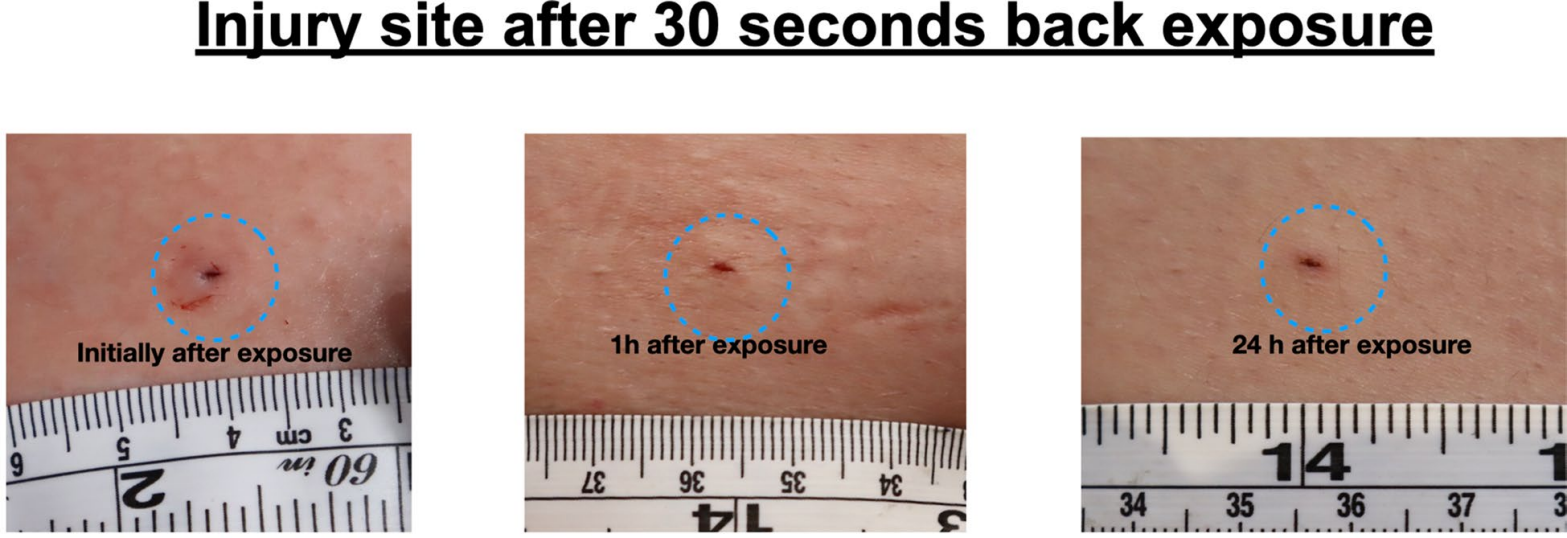
Injury site after 30 s back exposure with a T10

After removal of the dart electrodes, all wounds showed consistent morphology across all participants, characterized by a central probe penetration point. In some cases, a small surrounding area of blanching was noted, likely caused by localized vasoconstriction. No signs of electrical burns or other significant skin injuries were observed. There was no difference in wound morphology between front (Fig. 4) and back (Fig. 5) exposition.

Participants described the initial sensation of the exposure as a brief, pressure-like pain localized at the dart site. Following exposure, they reported mild discomfort in the affected area, likened to the sensation of a larger mosquito bite. At the 24-h follow-up, minor crusting (measuring a few millimeters in diameter) was observed at the probe penetration sites, with no additional signs of injury or morphological changes. Participants reported no lasting discomfort or adverse effects.

All subjects were instructed to report any worsening symptoms or complications within 48 h post-exposure. No such complaints were received from any participant during this period.

## Discussion

The mechanical injury potential of next-generation CEW darts is influenced by several factors, including dart velocity, shooting distance, impact location, tissue-specific variability, angle of impact, and body habitus. These biomechanical aspects of dart deployment onto the human body have been extensively addressed in previous studies [1]. However, the focus of this study was not on mechanical injuries but rather on the potential for electrical injuries during prolonged exposure.

Electrical shock trauma is known to produce complex injury patterns due to various frequency-dependent tissue-field interactions. In addition to thermal burns caused by Joule heating, mechanisms such as cell membrane permeabilization and electroconformational denaturation of macromolecules (e.g., proteins) have been identified as potential contributors to tissue damage, potentially leading to necrotic changes. Despite these theoretical risks, no macromorphological changes indicative of electrical injury were observed in any of the long-duration exposure sites examined in this study.

Consistent with prior research [2–4], dart penetration into human tissue typically results in perforation of the epidermis and the creation of a wound track through the dermis. Histological analysis in earlier studies has demonstrated an initial acute inflammatory response without evidence of vasculitis or thrombosis. As expected for minor wounds, these injuries transition to dry scabs within 24–48 h, showing no distinguishing characteristics that would identify them as CEW-related. The low infection risk associated with CEW darts further reduces the likelihood of wound healing complications [3]. After exposure, a primary wound care is sufficient, and no further wound-specific medical interventions are necessary [5].

Overall, the observed cutaneous injuries in this study appear to be attributable solely to the mechanical effects of dart penetration, with no evidence of electrical tissue damage. These wounds are expected to heal without scarring, further supporting the safety profile of the TASER 10 device during extended exposure.

## Limitations

This study focused exclusively on the electrical effects of prolonged TASER T10 exposure on human skin and did not analyze the ballistics or mechanical impact of the dart. While the number of experiments conducted was relatively small, the results demonstrated minimal variability. This consistency aligns with the well-established understanding that standardized electrical testing typically exhibits limited variability in fundamental parameters such as voltage, resistance, and current. Therefore, the findings of this study provide a reliable foundation for evaluating potential injury patterns associated with the TASER T10. Furthermore, the data presented here can serve as a basis for future research and more comprehensive evaluations.

## Conclusion

Our experiments demonstrated that there is no higher injury risk to the skin and underlying tissue during a long term exposure of 20–30 s to the waveform of the T10, compared to short term 5 s cycle.

### Key points

1. Injury potential of next gerneration CEW darts is primarily influenced by biomechanical factors.
2. The typical dart injury of CEW consists of an approx. 1 mm-large perforation of the epidermis and dermis.
3. Long-duration exposure of up to 30 seconds to CEW does not cause electrical injuries at the dart site.
4. After exposure, a primary wound care is sufficient to treat the dart injury.

## Data Availability

The datasets generated during and/or analyzed during the current study are available from the corresponding author on reasonable request.
